# Effects of a Heating System on Waste Plastic Pyrolysis in a Fixed-Bed Reactor

**DOI:** 10.3390/polym18212702

**Published:** 2026-11-01

**Authors:** Saša Papuga, Sergio Brutti, Stefano Vecchio Ciprioti, Omar Al Zeer

**Affiliations:** 1 Faculty of Technology, University of Banja Luka, 78000 Banja Luka, Bosnia and Herzegovina; 2 Department of Chemistry, Sapienza University of Rome, 00185 Rome, Italy; sergio.brutti@uniroma1.it; 3 Department of Basic and Applied Sciences for Engineering, Sapienza University of Rome, 00161 Rome, Italy; omar.alzeer@uniroma1.it

**Keywords:** plastic pyrolysis, fixed-bed reactor, heating mode, hot spots, pyrolysis-oil composition

## Abstract

Plastic pyrolysis performance depends not only on nominal reactor temperature but also on the actual temperature field established within the reacting bed and vapor phase. This study investigates how the heating-system configuration of a laboratory fixed-bed reactor affects product yields and pyrolysis-oil composition during waste polypropylene pyrolysis. Experiments were performed at 475 °C under N_2_ at 0.1 L min^−1^ for 60 min, comparing a single PID-controlled internal heater with a dual configuration using two independently controlled heating systems. Dual heating increased mean oil yield from 52.14 to 83.95% and reduced gas yield from 45.87 to 14.55%, while solid residue decreased from 1.99 to 1.50%. Temperature profiles showed pronounced overheating in the central vapor-phase region during single heating, whereas dual heating maintained a more homogeneous profile close to the set point. ATR-FTIR, ^1^H NMR, and elemental analysis further showed that the dual-heating oil was more aliphatic, with the aliphatic proton fraction increasing from 80.8 to 88.7% and the mean atomic H/C ratio increasing from 2.019 to 2.135. These results demonstrate that heating configuration can substantially alter both yield distribution and liquid-product chemistry even at the same nominal reactor temperature.

## 1. Introduction

Pyrolysis is a process of thermochemical decomposition of organic material in an inert atmosphere or environment with a very small amount of oxygen in order to suspend char formation [1]. During the pyrolysis of plastic materials, macromolecular chains of polymers are broken down into relatively smaller and simpler gaseous products, whereby a relatively small amount of solid residue remains in the form of ash in the reactor. Part of the gaseous or steam products is separated on the condensation system in the form of a liquid product, i.e., pyrolysis (pyrolytic) oil, while the non-condensable part of the product passes through the system, i.e., it is separated into the form of gaseous products. The products can be used as fuels, raw materials in the petrochemical industry or monomers [2,3,4,5]. Polymers such as polystyrene and polymethyl methacrylate during pyrolysis produce initial monomers, other monoaromatic compounds, along with other hydrocarbons, while polyethylene and polypropylene yield only 0–2% of initial monomers, and are generally transformed into other hydrocarbons [6,7,8]. Furthermore, valuable products like phenols and aromatics are obtained from thermal and catalytic pyrolysis of polycarbonates and high-impact polystyrene, respectively [9]. Thus, it is possible to say that pyrolysis is a waste-plastic recycling method that complements physical recycling. There is a consensus that pyrolysis might be a crucial technology in the years to come [10].

The process temperature is the most important parameter because it affects the amount of cracking of the polymer chains [11,12]. Low-temperature processes, generally up to 600 °C, are used to increase the relative proportion of the liquid phase, or pyrolytic oil, in the process products, while high-temperature processes, over 800 °C, are used if one wants to increase the volume of gas phase production [5,13]. At the same time, temperatures above 600 °C cause cyclic hydrocarbons, aromatics, coke formation and the dewaxing mechanism [14]. The relative share of individual phases in pyrolysis products, in addition to the applied temperature, is also determined by other process conditions such as reaction time, carrier gas flow, heating rate, product cooling rate, applied pressure, particle size of the starting raw material, as well as the nature of the raw material itself and the type of reactor [15,16,17,18,19]. Therefore, a large variation in the obtained results can be observed in the literature, both in terms of the yield of the pyrolysis process and the need for catalysts as well as in terms of the kinetics of the process and the quality of the pyrolysis products [8,12,20,21,22]. Recent studies have also demonstrated the effectiveness of catalytic pyrolysis for the valorization of complex and heterogeneous plastic wastes. Catalytic pyrolysis of marine plastic litter collected during a beach-cleanup campaign proved to be a valuable route for the production of hydrocarbon-rich oils and gases [23]. Similarly, coal-fly-ash-derived zeolites have been successfully employed for the catalytic pyrolysis of residual plastic waste, promoting the conversion of low-value polymer residues into liquid oils and valuable chemicals [24]. Additionally, this is supported by the fact that most research is based on thermogravimetric tests [25,26,27,28,29,30]. These tests are carried out on relatively small samples and most often in autoclaves, dedicated devices for conducting TGA, i.e., conditions in which there are no restrictions in terms of heat transfer to the raw material particles. In one study of the pyrolysis of synthetic polymers and waste plastics with samples of only 15–75 mg, it is stated that the results obtained should be applied with caution to industrial processes [31]. Because plastics have low thermal conductivity, efficient heat transfer is essential to minimize temperature gradients within the polymer bed and promote a more uniform temperature distribution during pyrolysis [11,32,33]. Namely, it is necessary to provide an appropriate temperature drop through the layer of raw material, which is especially pronounced if the heating of the reactor is done through the outer walls of the reactor. Usually, reactors with a fixed bed are of the electric furnace type, in which the raw material is heated externally through reactor walls [34]. In other words, depending on the type of pyrolysis system, there may be a difference in the temperature of the reactor itself and the processed raw material, so that, at higher temperatures, the overall reaction rate may be determined by the heat transfer rate rather than by the reaction kinetics [35]. Moreover, the layer temperatures are different in different layers [36]. The use of laboratory fixed-bed reactors has also been investigated for the pyrolysis of other waste streams. In a recent study, tire waste pyrolysis was performed in a laboratory-scale fixed-bed batch reactor, and suitable operating conditions in terms of reaction time, temperature, and inert-gas flow rate were identified to favor the production of pyrolysis oil [37].

In general, the temperature can be controlled either by the furnace temperature (usually a thermoprobe is located on or in the reactor wall) or by the bed temperature. This may certainly be one of the reasons for the difference in published results regarding the yield of pyrolysis products. This raises the question of which temperature best represents pyrolysis conditions in a fixed-bed reactor: the reactor-wall temperature or the temperature within a specific feedstock layer? Giving a direct answer to the question asked could be tricky. Namely, product yield can be, and indeed is, correlated with the temperature in the vapor zone, but at the same time, it is also correlated with the temperature in the liquid or molten plastic phase, in which the pyrolysis process also occurs. Basically, efficacy of reactions and product formation, alongside overall heat and mass transfer conditions, is also influenced by heat distribution within the molten mass [38]. The concept of severity factor might be applied to determine the effective or representative temperature of the pyrolysis reactor in the process of plastic pyrolysis, similar to the case of biomass pyrolysis [39] or biomass hydrothermal processing [40]. However, in this study, we applied a statistically grounded approach examining whether the heating method of a specific fixed-bed reactor affects the yield and distribution of plastic pyrolysis products, considering the different temperature profiles of the feedstock sample during different heating modes. The reactor examined in this study is heated by means of heaters located in the middle of the reactor. This design makes the entire system more energy efficient. The operation of this reactor has been proven through a series of published studies [11,18,19,37,41,42,43].

In a study by Darko et al. [44], it was proven that the improved heating control of a fixed-bed reactor with six independent heating zones leads to a higher pyrolytic-oil yield and more valuable quality, primarily in terms of viscosity. However, the paper itself does not show the temperature change inside the reactor, i.e., the different layers of material, so the heating control is based on one thermoprobe located in the center of the raw material layer.

In an earlier paper by Papuga et al. [19], it was shown that, during the operation of a reactor with a simple heating control system, based on the response of only one thermoprobe, local overheating still occurs in different layers of material. Therefore, it was suggested that an independent double-heating system, i.e., a heating system independently controlled by the responses to two thermoprobes, would lead to a higher oil yield. This was especially noticed in the study by Papuga et al. [18] in which, among other things, it was proven that the oil yield increases with higher reactor loading as a result of reduced local overheating in the central zone of the reactor. Thus, for this study, the tested reactor was further improved in terms of the possibility of additional reactor temperature control regarding the dual-heating method.

## 2. Materials and Methods

### 2.1. Description of the Pyrolysis Experiment

**Figure 1:**
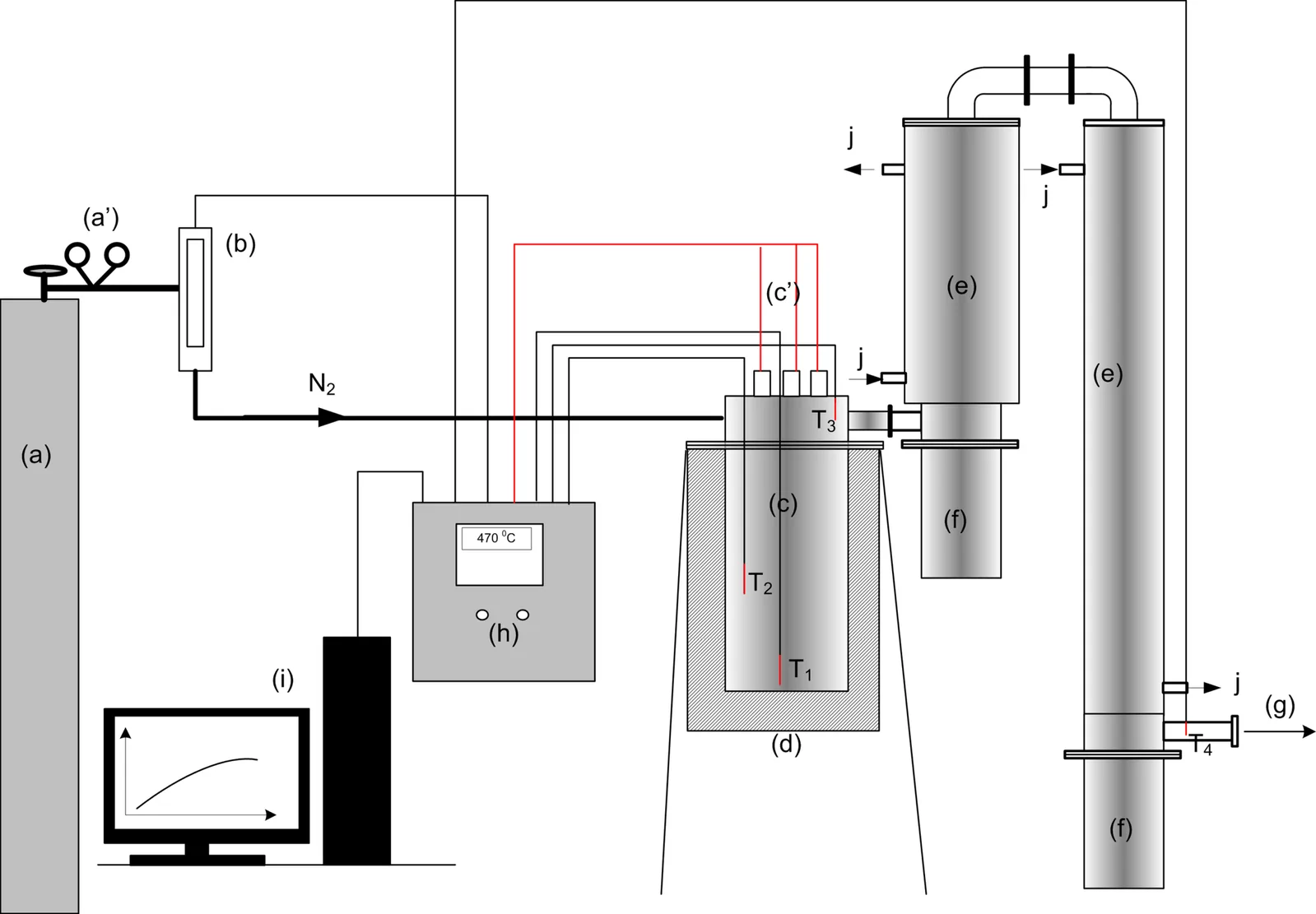
Block diagram of the experimental fixed-bed pyrolysis reactor: (**a**) cylinder with nitrogen gas; (**a’**) reduction valve; (**b**) gas mass flow meter; (**c**) the pyrolysis reactor vessel; (**c’**) heater electrical circuit; (**d**) thermal insulation; (**e**) steam condensation system; (**f**) separation system vessels for receiving condensate; (**g**) discharge of non-condensable gases in the gas washing system; (**h**) control box; (**i**) PC; (**j**) cooling water.

Waste polypropylene (PP), originating from food packaging, was used as a model sample. Sample preparation involved: washing, drying, crushing, and grinding on a laboratory mill. The average diameter of the sample particles was 1.40 mm, which was determined by granulometric analysis. The weight of each reactor feed was 100.00 g. The gas yield was calculated based on the difference between the total mass of the sample and the combined mass of the liquid and solid yields. Pyrolysis experiments were carried out in a laboratory fixed-bed reactor (Figure 1). A detailed description of the experimental technique and the fixed-bed reactor can be accessed from previous studies [19,39,40].

All pyrolysis experiments were carried out at a temperature of 475 °C, as an optimal value for complete conversation of reactor’s feed and according to the results of the previous studies in the same reactor configuration [11,18]. The carrier gas flow rate was 0.1 Ln min^−1^, and the reactor was inertized for 15 min before heating. The reactor was kept at 475 °C for 60 min. Nitrogen of 99.999% purity was used as the carrier gas, i.e., inertization atmosphere. For the purposes of this study, pyrolysis oil was defined as the condensed pyrolysis products, which basically consist of pyrolytic liquids and waxes, collected in the vessels for receiving condensate, marked as Figure 1f. A small amount of oily/waxy product, ranging from 0.5 to 1.5 g per experiment, remained stuck to the inner walls of the condensing system and lines, marked as Figure 1e. To avoid significant mass-closure uncertainty, this mass was collected mechanically, i.e., by cleaning the inner walls and lines with a cotton cloth, after which the difference in the mass of the cloth was determined. This difference in mass is added to the wax/oil yield collected in the condensing vessel indicated as Figure 1f.

### 2.2. Analytical Techniques

#### 2.2.1. ATR-FTIR Spectroscopy

ATR-FTIR spectra of pyrolysis oils A and T were recorded using a Bruker (Billerica, MA, USA) ALPHA II spectrometer equipped with a diamond attenuated-total-reflectance (ATR) accessory. Spectra were acquired over the 400–4000 cm^−1^ range using 64 scans at a spectral resolution of 4 cm^−1^. Two replicate measurements were collected for each oil. For comparative evaluation of selected functional-group regions, integrated band areas were normalized to the total C–H stretching region between 3100 and 2700 cm^−1^.

#### 2.2.2. ^1^H NMR Spectroscopy

Proton nuclear magnetic resonance (^1^H NMR) spectra were acquired on a Bruker (Billerica, MA, USA) Ascend 400 MHz spectrometer, using a 30° excitation pulse, a relaxation delay of 1.0 s, an acquisition time of approximately 4.0 s, and 16 scans. For each oil, 40 mg was fully dissolved in 0.6 mL of CDCl_3_. The residual CHCl_3_ resonance at 7.26 ppm was used as the chemical-shift reference. Measurements were performed at approximately 25 °C, and the experimental data were processed and integrated using MestReNova 15 (Mestrelab Research, Santiago de Compostela, Spain). The relative proton distribution was evaluated by semi-quantitative integration of the following chemical-shift regions: 0.4–1.0 ppm (predominantly aliphatic CH_3_), 1.0–1.8 ppm (aliphatic CH_2_/CH), 1.8–3.3 ppm (deshielded aliphatic hydrogens adjacent to unsaturated/aromatic environments), 4.5–6.5 ppm (olefinic hydrogens), and 6.5–9.0 ppm (aromatic hydrogens), excluding the residual CDCl_3_ resonance. This regional approach follows established ^1^H NMR characterization schemes for complex hydrocarbon and pyrolysis-oil matrices [45,46,47].

#### 2.2.3. Elemental Analysis

Elemental C, H, N and S determinations were performed using an EA 1110 CHNS-O (CE Instruments, Milan, Italy) analyzer with EAGER 200 data processing. The analytical sequence involved combustion of the sample at approximately 1000 °C; catalytic oxidation/reduction of the combustion products; gas-chromatographic separation of N_2_, CO_2_ and H_2_O; and thermal-conductivity detection. The estimated uncertainty for an individual C or H determination was ±0.3 wt%. Nitrogen and sulfur were not detected in the analyzed aliquots. Because the oils are volatile and compositionally heterogeneous, absolute C and H values were interpreted together with the atomic H/C ratio [48].

### 2.3. Experimental Design and Statistical Analysis

The experimental design and associated statistical testing, as tools for drawing conclusions about the hypothesis, were carried out using the Design-Expert 11 software package (Stat-Ease, Inc., Minneapolis, MN, USA).

**Figure 2:**
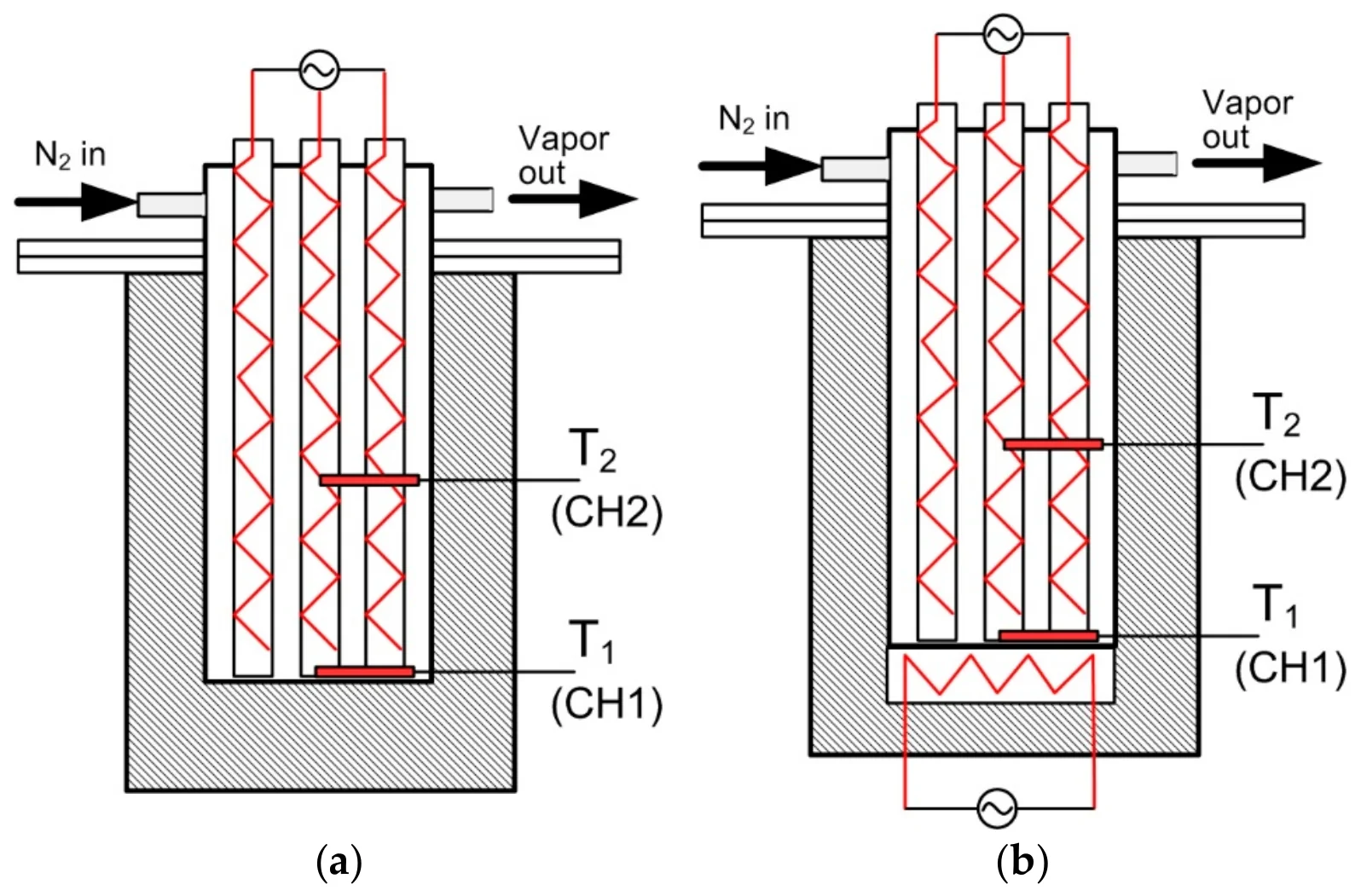
Cross-section of the reactor: (**a**) single-heating system, (**b**) dual-heating system. CH1 and CH2 indicate the bottom and middle sections of the reactor, respectively.

To test the hypothesis of the influence of the reactor heating mode, as an independent variable, on the yield and distribution of pyrolysis products, as dependent variables, a factorial experimental design was adopted. Heating mode (Factor A) was tested as a categorical parameter with two levels (treatments) of influence:

- Level 1 (single-heating mode)—heating of the reactor with the single PID-controlled heating system located inside of the reactor, in the middle of the feedstock bed (Figure 2a);
- Level 2 (dual-heating mode)—Simultaneous heating of the reactor with two PID independently controlled heating systems. The first heating system is located inside of the reactor (heating system defined as Level 1), and the second heating system is located on the outer bottom wall of the reactor (Figure 2b).

The PID control loop of the first heating system uses a thermoprobe located in the middle part of the reactor. The PID control loop of the second heating system uses a thermoprobe located at the bottom of the inner part of the reactor.

As dependent variables (measured responses), pyrolysis-oil yields (R1), gas yield (R2) and solid residue (R3) in the reactor were monitored. Each level of influence was tested through three replicate experiments. According to the adopted experimental design, this enabled a statistically reliable assessment of the tested treatment (Table 1). As Table 1 shows, the statistical power of the chosen experimental design to detect the effect of interest, relative to the influence of factor A on all three dependent variables, is significantly above the recommended minimum of 80%. In general, the statistical power of an experimental design should be at least 80% [49,50].

The estimated standard deviations in oil (R1), gas (R2) and solids yield were adopted as 0.67, 0.46 and 0.28%, respectively. Such low estimated values are based on experience from previous experiments on the same reactor and are certainly better for statistical power. The minimum changes that the design should detect as statistically significant were adopted at 2.5 in relation to R1 and R2 and 1% in relation to R3.

The statistical significance of the effect of Factor A on R1, R2, and R3 was tested in two ways:

1. Model F-test, or analysis of variance (ANOVA) of the hypothetical factorial model of influence, with the use of Fisher’s statistical test (F-test);
2. Fisher’s LSD test, i.e., pairwise comparison of treatment means, was used as additional confirmation. This test evaluates the null hypothesis that the treatment means are equal.

## 3. Results and Discussion

Table 2 shows the yields of pyrolysis oil (R1), pyrolysis gas (R2) and solid residue (R3) when applying different heating modes. As shown in Table 2, there are large variations in individual values of responses R1 and R2 depending on the applied treatment. The significance of these variations is further statistically approved by analyzing the variance of the hypothetical factorial model that describe the influence of Factor A (heating mode) on the responses in question, as well as by F-test (Table 3).

### 3.1. Statistical Testing

Table 3 shows that the model F-values, as well as the sum of squares for responses R1 and R2, are quite large compared to the model F-values for response R3. For the case of response R1, the model F-value of 6612.61 implies the model is not significant relative to the noise. There is only a 0.01% chance that an F-value this large could occur due to noise. For the case of response R2, the model F-value of 4983.06 implies the model is significant relative to the noise. There is less than 0.01% chance that an F-value this large could occur due to noise. For the case of response R3, the model F-value of 46.47 implies the model is significant relative to the noise. There is only a 0.24% chance that an F-value this large could occur due to noise. Analogous statements can be made based on the observation of *p*-values. Generally, *p*-values less than 0.0500 indicate model terms (Factor A) are significant. In this case, Factor A is a significant model term, with respect to yields of all three responses. Values greater than 0.1000 indicate the model terms are not significant. Therefore, it is possible to conclude that the mode of fixed-bed reactor heating has significant effect on the yield of pyrolysis products.

**Figure 3:**
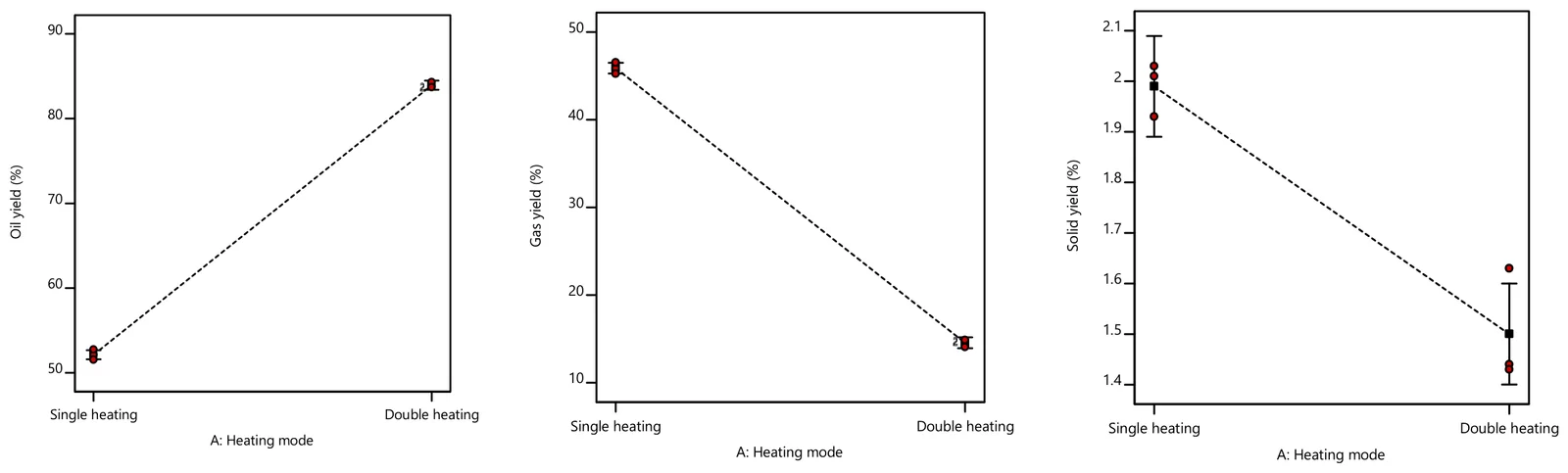
LSD I-beams pairwise comparison of the average response values (significance level: α = 0.05).

The above conclusions can be reached by comparing the corresponding I-beams on the graphs presented in Figure 3. The graphs show the average values of the dependent variables with regard to the appropriate treatment (single- or dual-heating mode), as well as the corresponding LSD intervals, i.e., intervals of 95% least significant differences (Fisher’s LSD test), which are represented by vertical interval lines (I-beams). In order for the two treatments to differ significantly in terms of impact on the response of interest, there must be no horizontal overlapping of the I-beams. All three graphs clearly show that the ranges of values included in the I-beams on the left side of a graph (bottom inertization) do not overlap with the corresponding I-beams on the right side of the graphs (top inertization). Thus, it is possible to conclude that there is a significant difference between two modes of fixed-bed reactor heating with regard to the yield of pyrolysis products.

### 3.2. Temperature-Profile Analysis

**Figure 4:**
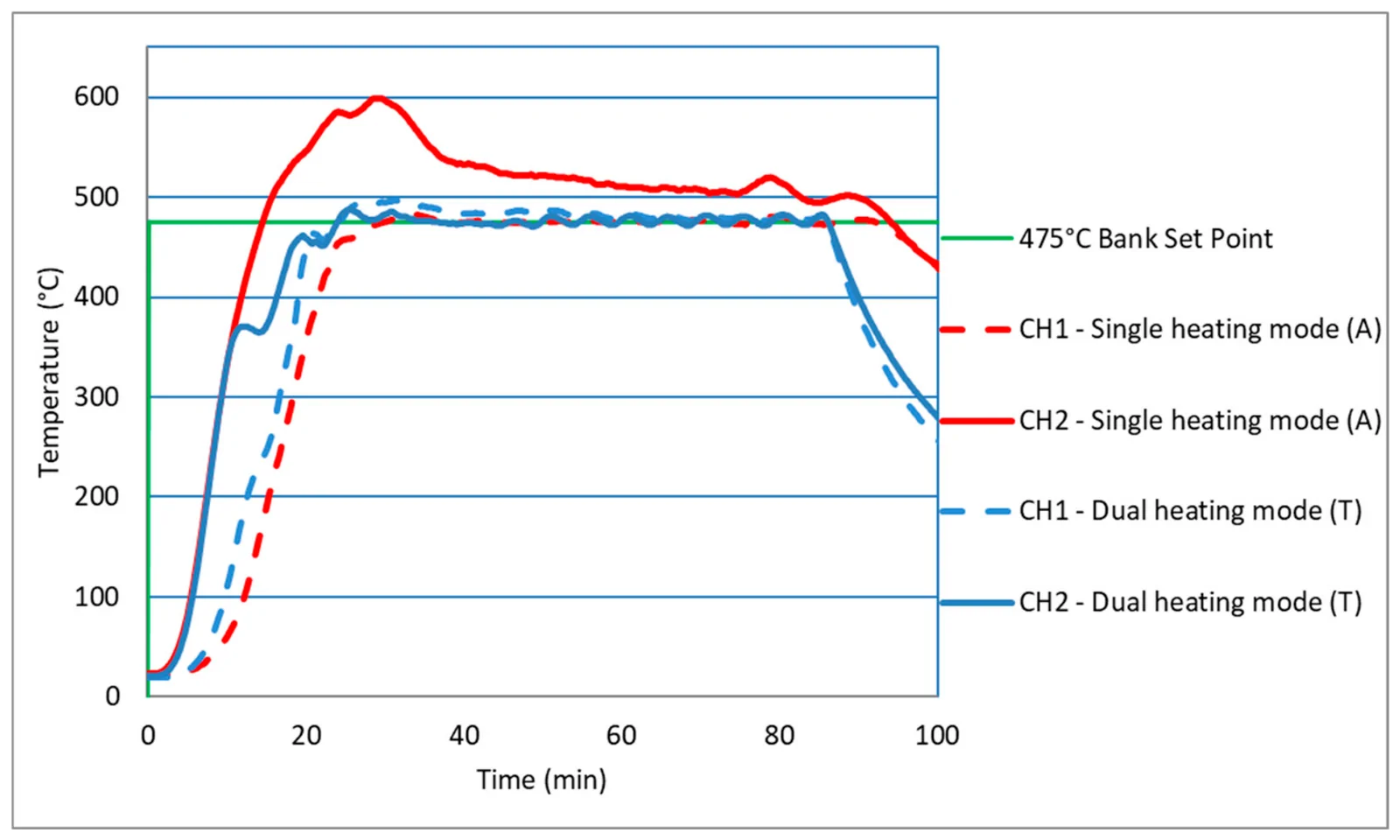
Temperature changes in the bottom (CH1) and middle (CH2) sections of the reactor during single- and dual-heating modes.

The significantly higher yield of pyrolytic oil in the dual-heating mode is attributed to the change in temperature inside the reactor (Figure 4), which shows that this particular fixed-bed reactor suffers from local overheating in its middle section, i.e., temperatures exceed the set/desired value (475 °C bank set point, in Figure 4). A somewhat similar behavior of the temperature field inside a fixed-bed reactor was also recorded in the study by Swamardika et al. [33] during the pyrolysis of PP waste, as well as in previous studies of this particular reactor system [19,43]. Local overheating of the middle section of the reactor is more pronounced in the case of single-heating mode compared to dual-heating mode. This occurrence leads to a more extensive cracking of volatile products from saturated aliphatic plastics, such as PP, and thus greater formation of non-condensable gases, i.e., a reduction in the yield of pyrolytic oil [44,51].

In general, higher pyrolysis temperatures promote the formation of gas at the expense of liquid products. Temperatures from 400 to 550 °C maximize liquid oil and wax yields, while temperatures above 600 °C shift yields toward C1–C4 gases and olefins through secondary cracking [51]. From Figure 4, it is quite obvious that, in the single-heating mode, most of the time, the middle section of the reactor is well above 500 °C, while the lower part of the reactor, where the plastic melts, decomposes, evaporates and enters the pyrolysis reaction, almost ideally maintains the desired temperature of 475 °C. In the case of dual-heating mode, such a significant amount of overheating does not occur, and most of the time, the reactor is in the desired temperature range.

The above statement about excessive cracking of volatile products during a single-heating mode is further supported by the chemical characterization of pyrolysis oils. Namely, it is well known that the distribution of pyrolysis-oil products is strongly controlled by temperature. Increasing the pyrolysis temperature promotes thermal cracking, free radical reactions, depolymerization and polycondensation, creating aromatic and unsaturated compounds [52].

Lower temperatures (300 to 400 °C) retain or yield aliphatic structures, whereas high temperatures (>500 °C) break down aliphatic chains, eliminate hydrogen and oxygen, and form stable aromatic ring networks [53]. Furthermore, it is worth noting that actual plastic materials may contain varying amounts of various additives, such as inorganic fillers, pigments, and flame retardants, depending on their intended use. In general, these additives can act as in situ catalysts in the pyrolysis process of various types of plastic [54,55]. Presence of additives, such as magnesium silicate-based fillers, in waste polypropylene can lead to a higher content of aromatic hydrocarbons in the pyrolytic oil compared to the virgin polymer [56]. However, for this research, we used polypropylene from food packaging, which typically contains no mineral fillers or only very small amounts, so we can assume that the influence of such fillers on the pyrolysis mechanism and the quality of the pyrolytic oil, if any, would be quite limited. Furthermore, the same sample was used in every experiment, meaning that any potential impact from a specific concentration of fillers would remain consistent across all experiments. Also, as we have already proven, the two analyzed heating modes result in different temperature profiles in the reactor, which, quite naturally, could lead to a different behavior of the fillers that could acts as an in situ catalyst. Thus, if there is any influence from the filler, it would be a consequence of varying temperatures within the reactor, or in other words, it could be considered in relation to, and alongside, the heating effect.

The results of chemical characterization of oils obtained during pyrolysis under single-heating mode conditions (sample A) and during pyrolysis under dual-heating mode conditions (sample T) are presented in the following section.

### 3.3. Chemical Characterization of the Pyrolysis Oil

The chemical composition of the liquid products obtained under the two reactor-heating configurations was investigated by ATR-FTIR spectroscopy, ^1^H NMR spectroscopy, and elemental analysis. Two pyrolysis-oil samples were selected for the comparative chemical characterization, each corresponding to one of the heating configurations investigated throughout this study. Sample A was the liquid product obtained from the pyrolysis of waste polypropylene under the single-heating configuration, whereas sample T was obtained from the same polypropylene feedstock under the dual-heating configuration. In both cases, the nominal operating conditions were those described above, namely a reactor temperature of 475 °C, a nitrogen flow rate of 0.1 L min^−1^, and a reaction time of 60 min. Thus, A and T provide a direct comparison between the liquid products generated under the two heating modes while keeping the feedstock and nominal pyrolysis conditions unchanged. The sample labels A and T are used hereafter throughout the spectroscopic and elemental analyses.

FTIR spectroscopy and ^1^H NMR spectroscopy are widely used as complementary techniques for the characterization of complex pyrolysis oils and hydrocarbon mixtures. They provide information on functional-group distributions and on the relative abundance of aliphatic, olefinic and aromatic environments [45,46,47].

As discussed in the preceding section, the single-heating configuration generated pronounced local overheating in the middle part of the fixed-bed reactor, whereas the dual-heating configuration produced a considerably more homogeneous temperature profile close to the nominal set point of 475 °C. Since the feedstock and nominal process conditions were otherwise unchanged, the chemical characterization of A and T was used to assess whether the different thermal histories experienced by the pyrolysis vapors were also reflected in the composition of the condensed liquid products.

#### 3.3.1. ATR-FTIR Analysis

The ATR-FTIR comparison was performed using relative spectral indices. For each diagnostic spectral interval, the integrated absorbance of the selected band was divided by the integrated area of the broad C–H stretching region between 3100 and 2700 cm^−1^, which was used as an internal spectral reference. This treatment reduces the influence of differences in overall spectral intensity and allows the relative contribution of the same functional-group region to be compared between oils A and T.

**Figure 5:**
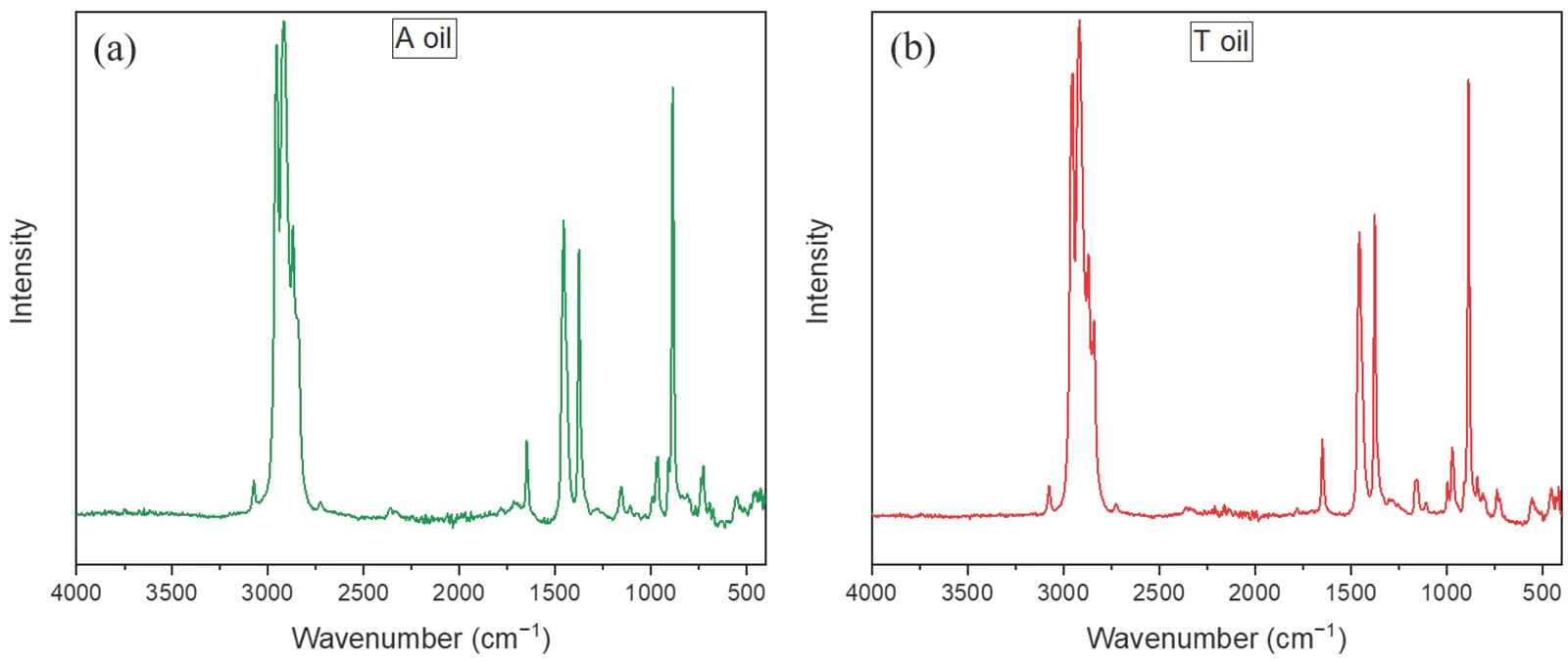
ATR-FTIR spectra of pyrolysis oils obtained under (**a**) single-heating conditions (oil A) and (**b**) dual-heating conditions (oil T).

I_region_ = A_region_/A_3100–2700_

where I_region_ is the dimensionless normalized FTIR index, A_region_ is the integrated absorbance of the selected wavenumber interval, and A_3100–2700_ is the integrated absorbance of the reference C–H stretching envelope. The results and spectra are shown in Table 4 and Figure 5.

Both oils are dominated by intense C–H stretching absorptions in the 2950–2850 cm^−1^ region, confirming their predominantly aliphatic hydrocarbon character. This assignment is consistent with previous FTIR studies of pyrolysis oils. Yan et al. assigned bands near 2957, 2922, and 2870 cm^−1^ to methyl and methylene stretching vibrations in oils from thermal cracking of PP and LDPE, while Banar et al. likewise associated the 2800–3000 cm^−1^ region with alkane C–H stretching [45,47]. The carbonyl index was very small for both samples (0.0064 for A and 0.0053 for T), corresponding to only 0.64 and 0.53% of the respective C–H reference areas. The weak carbonyl-region absorption may originate from prior oxidative ageing of the waste PP and oxygen-containing additives or contaminants, which could yield oxygenated products even under nitrogen. Contributions from trace oxygen or subsequent oil oxidation cannot be excluded, and the available data do not establish a unique origin. A clearer difference was observed in the 1395–1365 cm^−1^ CH_3_ symmetric-bending region, where the normalized index increased from 0.0741 for A to 0.0885 for T. This increase indicates a larger relative methyl contribution in oil T.

Overall, the ATR-FTIR results indicate that both oils retain a predominantly aliphatic hydrocarbon character, while the relative distribution of specific functional groups differs between the two heating configurations. In particular, the higher CH_3_-related contribution observed for oil T is consistent with a more pronounced aliphatic character under dual-heating conditions.

#### 3.3.2. ^1^H NMR Analysis

**Figure 6:**
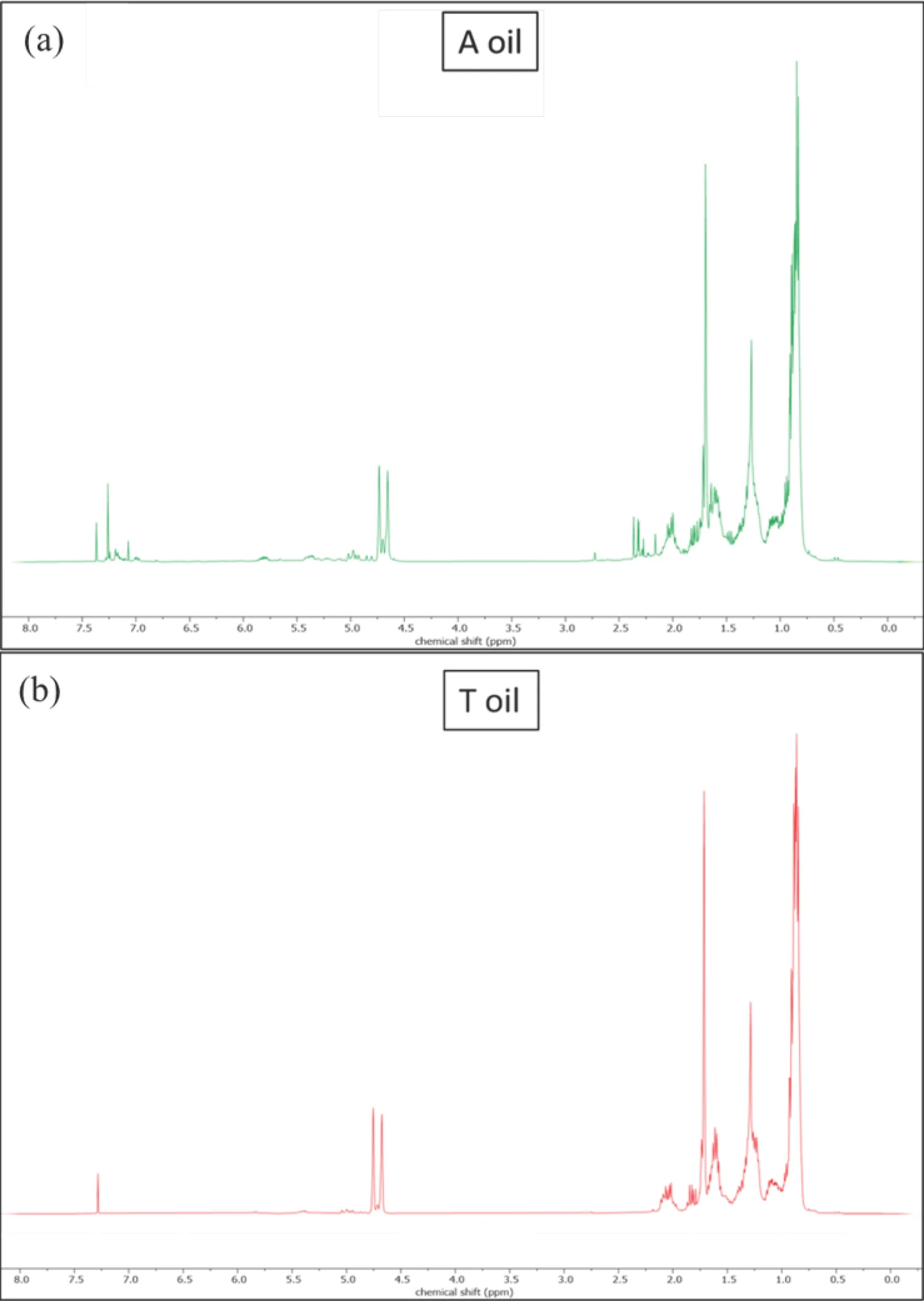
^1^H NMR spectra of pyrolysis oils obtained under (**a**) single-heating conditions (oil A) and (**b**) dual-heating conditions (oil T). CDCl_3_ was used as solvent, and the residual solvent signal at 7.26 ppm was excluded from regional integration.

The ^1^H NMR spectra were evaluated by direct regional integration of the proton signal. Five chemical-shift intervals were defined on the basis of established pyrolysis-oil assignments: 0.4–1.0 ppm for highly shielded aliphatic CH_3_ protons, 1.0–1.8 ppm for aliphatic CH_2_/CH protons, 1.8–3.3 ppm for deshielded aliphatic protons located near unsaturated or aromatic functionalities, 4.5–6.5 ppm for olefinic protons, and 6.5–9.0 ppm for aromatic protons. The 7.25–7.27 ppm interval containing the residual CHCl_3_ resonance was excluded from the aromatic integral. Banar et al. use closely comparable hydrogen-type regions for pyrolytic oils, while Yan et al. [47] report analogous aliphatic, vinylic, and aromatic regions for PP- and LDPE-derived oils.

fi (%) = 100 × A_i_/ΣA_j_

where A_i_ is the integrated ^1^H NMR area of region i and ΣA_j_ is the sum of the selected proton-region integrals after exclusion of the solvent resonance. The reported values therefore represent the percentage distribution of observed proton environments. Results and spectra are shown in Table 5 and Figure 6.

Both oils were predominantly aliphatic, but a clear difference was observed between the two heating configurations. For sample A, the 0.4–1.0 ppm and 1.0–1.8 ppm regions accounted for 37.6 and 43.2% of the integrated proton signal, respectively, resulting in a total aliphatic contribution of 80.8%. Deshielded aliphatic hydrogens represented 9.8%, olefinic hydrogens represented 7.9%, and aromatic hydrogens represented 1.5%.

In contrast, oil T showed a markedly greater aliphatic contribution. The CH_3_ and CH_2_/CH regions accounted for 43.5 and 45.2%, respectively, giving a total aliphatic fraction of 88.7%. At the same time, the deshielded aliphatic contribution decreased to 5.2%, and the olefinic fraction decreased to 6.1%. The aromatic region integral for oil T corresponded to 0.0% at the reported precision, compared with 1.5% for oil A.

The change from single to dual heating therefore increased the total aliphatic hydrogen fraction from 80.8 to 88.7%, while the combined deshielded, olefinic and aromatic contribution decreased from 19.2 to 11.3%. The olefinic fraction decreased from 7.9 to 6.1%, and the aromatic contribution decreased from 1.5% to 0.0% at the reported precision. These results are consistent with the predominantly aliphatic/olefinic products expected from polyolefin pyrolysis. In particular, Yan et al. [47] reported significant formation of unsaturated hydrocarbons during PP thermal cracking and identified intramolecular hydrogen transfer and β-scission as major degradation pathways.

The greater relative contribution of deshielded, olefinic and aromatic proton environments in oil A is consistent with the more severe thermal history experienced by the evolving vapors under single-heating conditions. Selective loss of light components to the non-condensed fraction, or differences in their recovery, may also contribute to the observed distributions.

#### 3.3.3. Elemental Analysis

Elemental analysis was evaluated at the level of individual aliquots before calculation of descriptive statistics. Nitrogen and sulfur were not detected in any A or T aliquot. The atomic hydrogen-to-carbon ratio was calculated separately for each determination from the measured carbon and hydrogen mass fractions:

H/C = (w_H_/1.008)/(w_C_/12.011)

where w_H_ and w_C_ are the measured hydrogen and carbon mass fractions. Calculating H/C for each aliquot preserves the covariance of the paired C and H measurements and provides a more chemically meaningful descriptor than calculating a ratio from independently averaged C and H values. The residual quantity 100 − C − H was treated only as an unassigned mass balance; it was not interpreted as a direct oxygen determination because the method did not directly quantify oxygen and the oils may contain volatile or suspended fractions.

The results of the elemental analysis are reported in Table 6 and Table 7. Table 6 summarizes the individual C, H, N, and S determinations and the corresponding atomic H/C ratios for each aliquot, whereas Table 7 presents the descriptive statistics for oils A and T.

The mean atomic H/C ratio increases from 2.019 ± 0.029 for A to 2.135 ± 0.013 for T. Importantly, the observed H/C ranges do not overlap: all T aliquots fall between 2.126 and 2.150, whereas all A aliquots fall between 1.985 and 2.052. The absolute C and H values are more dispersed for A than for T, particularly for carbon. This dispersion should be interpreted in the context of the volatile and compositionally heterogeneous nature of pyrolysis oils, for which subsampling can contribute to analytical variability. The higher H/C ratio of T agrees with the ^1^H NMR result: T has the largest aliphatic proton fraction and the smallest combined deshielded, olefinic, and aromatic contribution. Conversely, A has a lower H/C ratio and the larger relative contribution of unsaturated/aromatic hydrogen environments.

Under dual-heating conditions, the total aliphatic proton contribution rises from 80.8 to 88.7%, and the atomic H/C ratio rises from 2.019 ± 0.029 to 2.135 ± 0.013. In parallel, the olefinic and aromatic proton contributions decrease. These changes are consistent with a lower effective thermal severity of the vapor phase when the central overheating observed in the single-heating configuration is suppressed.

## 4. Conclusions

This study demonstrates that the heating-system configuration of a fixed-bed reactor can substantially affect both the product distribution and the chemical characteristics of the liquid products obtained during waste polypropylene pyrolysis, even when the nominal operating conditions are kept unchanged. Switching from a single- to a dual-heating configuration increased the mean pyrolysis-oil yield from 52.14 to 83.95%, while the gas yield decreased from 45.87 to 14.55%, and the solid residue decreased from 1.99 to 1.50%. The influence of the heating mode on all three product yields was statistically significant, as confirmed by ANOVA and Fisher’s LSD analysis.

Analysis of the internal temperature profiles provides a physical explanation for these differences. Under single-heating conditions, pronounced overheating developed in the central region of the reactor, whereas the dual-heating configuration maintained a considerably more homogeneous temperature profile close to the nominal set point of 475 °C. The greater thermal severity experienced by the evolving vapors under single heating is consistent with enhanced secondary cracking and, consequently, with the greater formation of non-condensable products.

The chemical characterization of the representative pyrolysis oils further supports this interpretation. Both oils were predominantly aliphatic; however, ATR-FTIR showed a greater relative CH_3_ contribution for the oil obtained under dual heating. More clearly, ^1^H NMR showed that the total aliphatic proton fraction increased from 80.8% under single heating to 88.7% under dual heating, while the combined deshielded, olefinic, and aromatic contribution decreased from 19.2 to 11.3%. Consistently, the mean atomic H/C ratio increased from 2.019 ± 0.029 to 2.135 ± 0.013. Together, these results indicate that suppression of the central reactor hotspot reduces the effective thermal severity experienced by the vapor phase and produces a more strongly aliphatic liquid product.

Overall, the results demonstrate that nominal reactor temperature alone is insufficient to fully describe fixed-bed plastic-pyrolysis conditions. A similar effect would be maintained at other temperatures typical for pyrolysis of plastics, as well as at different masses of raw material, especially with a higher reactor load. Namely, a similar behavior—in terms of overheating of the central part of the reactor with a fixed bed in single-heating mode operation—has already been demonstrated [18,19,43]. One could expect a similar effect with other polymers, such as HDPE or PS; although the pyrolysis mechanism itself differs somewhat compared to the PP studied, the fundamental conditions regarding heat transfer and the overheating effect remain the same. The spatial temperature distribution and the thermal history of the evolving vapors must also be considered when interpreting product yields and oil composition. Improved control of the reactor temperature field can therefore simultaneously increase the recovery of condensable products and modify their chemical characteristics, providing a relevant design parameter for improving the reproducibility and performance of fixed-bed waste-plastic pyrolysis processes.

## Figures and Tables

**Table 1:** Statistical power of experimental design.

		Diff. to Detect *	Est. Std. Dev. **	Delta/Sigma ***	Power ****
Responses	Units	Delta (“Signal”)	Sigma (“Noise”)	(Signal/Noise Ratio)	(for Factors A)
Oil yield (R1)	%	2.5	0.67	3.73134	91.9
Gas yield (R2)	%	2.5	0.46	5.43478	99.7
Solid yield (R3)	%	1	0.28	3.57143	89.7

* Diff. to Detect (Difference to Detect) or Delta “Signal” is the minimum change in the response considered large enough to be a practical change. ** Est. Std. Dev. (Estimated Standard Deviation) or Sigma “Noise” is an estimate for the run-to-run standard deviation of the response. *** The Delta/Sigma (Signal/Noise) ratio is used to determine the probability of detecting an effect. **** Power is reported at a 5.0% alpha level to detect the specified signal/noise ratio.

**Table 2:** Yield of pyrolysis products (R1, R2, R3) with different heating modes of fixed-bed reactors.

**Factor A**	**Response 1**	**Response 2**	**Response 3**
Heating Mode	Oil Yield (%)	Gas Yield (%)	Solid Yield (%)
Single heating	52.11	45.86	2.03
Single heating	51.55	46.52	1.93
Single heating	52.75	45.24	2.01
Arithmetic mean	52.14	45.87	1.99
Standard error	0.2766	0.43137	0.0358
Dual heating	83.84	14.72	1.44
Dual heating	84.30	14.07	1.63
Dual heating	83.70	14.87	1.43
Arithmetic mean	83.95	14.55	1.50
Standard error	0.2766	0.3137	0.0508

**Table 3:** ANOVA for selected factorial models (Factor A influence).

Factor A	Response R1	Response R2	Response R3
Sum of Squares	1517.81	1471.41	0.3601
Pure Error	0.9181	1.18	0.0310
Cor Total *	1518.73	1472.59	0.3911
Model F-value	6612.61	4983.06	46.47
*p*-value	<0.0001	<0.0001	0.0024

* Corrected total sum of squares.

**Table 4:** Normalized ATR-FTIR indices for oils A and T. Each index is the integrated area of the indicated spectral region divided by the integrated 3100–2700 cm^−1^ C–H reference area.

Region (cm^−1^)	Main Assignment	Oil A	Oil T	A → T Trend
1750–1680	Carbonyl-containing species	0.0064	0.0053	slight decrease
1680–1600	C=C-containing/overlapping unsaturated region	0.0250	0.0317	increase
1475–1430	CH_2_/CH_3_ deformation	0.1647	0.1736	slight increase
1395–1365	CH_3_ symmetric bending	0.0741	0.0885	increase
915–900	Terminal olefins, RCH=CH_2_	0.0142	0.0145	unchanged
892–882	Terminal olefins, R_2_C=CH_2_	0.0706	0.0807	increase
870–650	Fingerprint/out-of-plane region	0.0463	0.0667	increase

**Table 5:** ^1^H NMR proton distribution of oils A and T obtained by regional integration.

^1^H Region (ppm)	Assignment	Oil T (%)	Oil A (%)	Interpretation
0.4–1.0	Aliphatic CH_3_	43.5	37.6	higher methyl contribution in T
1.0–1.8	Aliphatic CH_2_/CH	45.2	43.2	slightly higher in T
1.8–3.3	Deshielded aliphatic	5.2	9.8	markedly lower in T
4.5–6.5	Olefinic protons	6.1	7.9	lower in T
6.5–9.0	Aromatic protons	0.0	1.5	apparently absent in T
Total aliphatic (0.4–1.8 ppm)		88.7	80.8	higher in T
Deshielded + olefinic + aromatic		11.3	19.2	lower in T

**Table 6:** Individual elemental analysis determinations for oils A and T.

Oil	Rep.	C (wt%)	H (wt%)	S	N	C + H (wt%)	100 − C − H	H/C
**A**	1	83.35	14.04	ND	ND	97.39	2.61	2.007
**A**	2	73.47	12.65	ND	ND	86.12	13.88	2.052
**A**	3	78.00	13.29	ND	ND	91.29	8.71	2.030
**A**	4	82.24	13.70	ND	ND	95.95	4.05	1.985
**T**	1	80.68	14.56	ND	ND	95.24	4.76	2.150
**T**	2	79.56	14.20	ND	ND	93.75	6.25	2.126
**T**	3	81.73	14.61	ND	ND	96.34	3.66	2.130

ND = not detected. The 100 − C − H column is an unassigned balance and must not be equated directly with oxygen content.

**Table 7:** Descriptive elemental analysis summary for oils A and T.

H/C Mean ± SD	H Mean ± SD (wt%)	C Mean ± SD (wt%)	*n*	Oil
2.019 ± 0.029	13.42 ± 0.60	79.27 ± 4.50	4	A
2.135 ± 0.013	14.45 ± 0.22	80.66 ± 1.09	3	T

## Data Availability

The original contributions presented in this study are included in the article. Further inquiries can be directed to the corresponding authors.
